# A comparative study of MLP and LSTM neural networks for shale gas production prediction based on numerical simulation data

**DOI:** 10.1371/journal.pone.0336782

**Published:** 2025-11-13

**Authors:** Xiaoou Fei, Man Ye, Zhou Du, Haibin Miao

**Affiliations:** 1 School of Green Mining and Resource Engineering, Liaoning Petrochemical University, Fushun, Liaoning, China; 2 School of Mechanics and Engineering, Liaoning Technical University, Fuxin, Liaoning, China; 3 China Coal Technology and Engineering Group Shenyang Research Institute, Fushun, Liaoning, China; 4 State Key Laboratory of Disaster Prevention and Ecology Protection in Open-pit Coal Mines, Fushun, Liaoning, China; Wadia Institute of Himalayan Geology, INDIA

## Abstract

Accurate prediction of shale gas production is essential for optimizing reservoir development and improving production efficiency. In this study, a numerical simulation model was first developed to systematically calculate daily shale gas production under various engineering parameter combinations, thereby establishing a comprehensive production prediction database. Two types of deep learning models—multi-layer perceptron (MLP) and long short-term memory (LSTM) neural networks—were then constructed to predict daily shale gas production. Comparisons with actual production data for three representative scenarios revealed that the MLP model achieved relative errors of 2.43%, 6.36%, and 4.16%, while the LSTM model achieved superior accuracy with relative errors of 0.42%, 1.1%, and 0.98%. The LSTM network’s gating mechanisms effectively captured the long-term dependencies in shale gas production data, making it more suitable for complex multi-scale dynamic modeling compared to the feedforward MLP. These results demonstrate the excellent generalization capability and engineering applicability of deep learning techniques, particularly LSTM networks, for enhancing shale gas production forecasting and supporting the efficient development of unconventional gas reservoirs.

## 1. Introduction

The efficient development of unconventional tight shale reservoirs has become a crucial strategy for optimizing global energy structures and ensuring energy security. Since the first hydraulic fracturing operation by Pan American Petroleum in the Hugoton field in 1947, continuous advancements in horizontal drilling and multi-stage, multi-cluster hydraulic fracturing have significantly enhanced the economic viability of shale gas extraction in low-permeability, complex reservoirs, driving the rapid expansion of the shale gas industry [1–3]. However, the pronounced heterogeneity of shale reservoirs, multi-scale fracture networks, and complex geological structures continue to pose major challenges to accurately predicting single-well productivity. These challenges are largely attributed to the spatial variability of reservoir physical properties, strong stress interference between fractures, and the complex dynamic evolution of coupled multi-physical fields, which often result in substantial discrepancies and uncertainties in existing prediction approaches.

Currently, shale gas production prediction techniques primarily include decline curve analysis (DCA) based on production history data and numerical simulation methods grounded in physical mechanisms. DCA methods (e.g., Arps [4], PLE [5], SEPD [6], Duong [7]) are widely used for conventional tight reservoirs due to their simplicity and computational efficiency. However, their empirical assumptions fall short in fully capturing the intricate flow mechanisms in shale gas reservoirs. In contrast, numerical simulation approaches leverage multiphase flow theories to construct dual-porosity, multi-porosity, and complex fracture network models, systematically incorporating fracture geometry, gas adsorption–desorption, non-Darcy flow, and fracture network interactions. These models offer high-precision characterization of the stimulated reservoir volume (SRV) and production dynamics, particularly in simulating the cooperative expansion of complex fracture networks under multi-cluster hydraulic fracturing [8–15]. Nevertheless, when faced with high-dimensional input parameters and the need for real-time, scenario-specific recalibrations, numerical simulations can become computationally demanding and sensitive to model assumptions and parameter selections, limiting their broader applicability and operational flexibility.

As shale gas development enters an era of precision management and intelligent optimization, data-driven deep learning methods are increasingly recognized for their superior feature learning and high-dimensional nonlinear mapping capabilities. Multi-layer perceptron (MLP) neural networks excel in capturing complex multivariate relationships, while long short-term memory (LSTM) networks, with their gated architecture, are particularly effective in modeling long-term dependencies and dynamic variations in time-series data. LSTM has shown promise in predicting shale gas production decline trends, production response curves, and dynamic multi-scale production patterns under complex operating conditions, outperforming traditional feedforward networks [16–18]. However, the integration of deep learning models with physically informed numerical simulation data and the systematic evaluation of their comparative performance remain underexplored.

In this context, this study focuses on the intelligent prediction of shale gas production in multi-stage, multi-cluster hydraulic fracturing scenarios. A comprehensive shale gas production database was constructed using numerical simulation across various engineering parameter combinations. Two deep learning models—MLP and LSTM—were then developed to predict shale gas production. By systematically comparing the accuracy and generalization of these models, this study aims to elucidate their respective strengths and applicability, providing scientific support and practical guidance for the intelligent and efficient development of unconventional shale gas reservoirs.

## 2. Numerical simulation of multi-stage, multi-cluster hydraulic fracturing in shale reservoirs using horizontal wells

### 2.1. Research object

**Fig 1:**
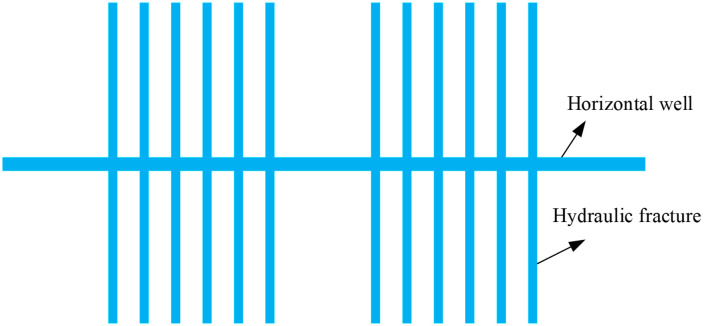
Schematic diagram of a multi-stage, multi-cluster hydraulically fractured horizontal well in a shale gas reservoir.

Fig 1 presents a numerical model of a horizontally drilled well in a shale gas reservoir, featuring two fracturing stages and six fracture clusters. The model primarily consists of the horizontal well section and multiple hydraulic fractures. High-pressure fracturing fluid is injected into the wellbore to generate a complex fracture network within the low-permeability shale formation, thereby enhancing gas flow capacity. Shale gas is released from the matrix, migrates through the induced fractures, and is eventually produced through the wellbore.

This model is designed to simulate the coupled mechanisms of fracture propagation and gas transport during hydraulic fracturing. Additionally, it serves as a source of high-quality synthetic data for training and validating the deep learning models developed in this study.

### 2.2. Constitutive model

In this study, a numerical model is developed to predict the production performance of multi-stage hydraulically fractured horizontal wells in a closed shale gas reservoir. The model is built upon the following fundamental assumptions:

(1) Hydraulic fractures are symmetric bi-wing structures and are perpendicular to the horizontal wellbore axis.
(2) The reservoir temperature remains constant throughout the production period.
(3) Gas adsorption behavior follows the Langmuir monolayer adsorption theory.
(4) A constant bottom-hole pressure production regime is applied, and gas enters the wellbore exclusively through the fracture system, with no direct matrix flow contribution considered.

The gas transport in the shale matrix system is governed by the following continuity equation:


$$-\nabla \cdot \left(\rho_{g}u_{m}\right)=\frac{\partial \left(\rho_{g}\varphi_{m}\right)}{\partial t}+\frac{\partial \left[\left(1-\varphi_{m}\right)\rho_{r}n_{a}M\right]}{\partial t}$$ (1)

In this equation, $\rho_{g}$ is the gas density (kg/m³), $u_{m}$ is the gas velocity within the matrix system (m/s), $\varphi_{m}$ represents the porosity of the shale matrix, $\rho_{r}$ is the rock density (kg/m³), $n_{a}$ is the absolute amount of adsorbed gas per unit mass of rock (mol/kg), and *M* is the molar mass of the gas (kg/mol). The first term on the right-hand side accounts for gas accumulation in the pore space, while the second term captures the variation in adsorbed gas over time.

The gas flow velocity in the shale matrix system can be described by Darcy’s law as:


$$u_{m}=-\frac{k_{m}}{\mu }\nabla p$$ (2)

Where $k_{m}$ is the apparent permeability of the shale matrix (m^2^), *μ* is the gas viscosity (Pa ⋅ s) and *p* is the pore pressure.

During shale gas production, changes in reservoir stress alter the matrix porosity and permeability. These pressure-dependent properties are given by:


$$\varphi_{m}=\varphi_{m0}{\left(\frac{p_{c}-p}{p_{0}}\right)}^{-q_{p}}$$ (3)

$$k_{m}=k_{m0}{\left(\frac{p_{c}-p}{p_{0}}\right)}^{-s_{k}}$$ (4)

where $\varphi_{m0}$ and $k_{m0}$ are the reference porosity and permeability under a reference pressure $p_{0}$ (Pa), $p_{c}$ is the confining pressure (Pa), $q_{p}$ and $s_{k}$ are the pressure sensitivity coefficients of porosity and permeability, respectively.

In the fracture network, gas transport is governed by the following continuity equation:


$$w_{f}\frac{\partial \left(\rho_{g}\varphi_{f}\right)}{\partial t}+\nabla \cdot \left(w_{f}\rho_{g}u_{f}\right)=0$$ (5)

where $u_{f}$ is the gas velocity in the fracture system (m/s), $\varphi_{f}$ is the fracture porosity, and $w_{f}$ is the fracture width (m).

The gas flow in the fracture system satisfies Darcy’s law, which can be expressed as:


$$u_{f}=-\frac{k_{f}}{\mu }\nabla p$$ (6)

where $k_{f}$ is the fracture permeability, with units of m².

During shale gas production, as reservoir pressure continuously declines, fractures gradually undergo compression. The fracture compressibility can be expressed as:


$$c_{f}=-\frac{\text{1}}{\varphi_{f}}\frac{\partial \varphi_{f}}{\partial \sigma_{e}}$$ (7)

where $\sigma_{e}$ is the effective stress (Pa).

Integrating both sides of Eq. (7) yields:


$$\int_{\sigma_{e0}}^{\sigma_{e}}c_{f}\partial \sigma_{e}=-\int_{\varphi_{f0}}^{\varphi_{f}}\frac{\text{1}}{\varphi_{f}}\partial \varphi_{f}$$ (8)

where $\sigma_{e0}$ is the initial effective stress (Pa) and $\varphi_{f0}$ is the initial fracture porosity.

The evolution of the fracture porosity with pressure can be described by:


$$\varphi_{f}=\varphi_{f0}e^{-c_{f}\left(p_{ini}-p\right)}$$ (9)

where $p_{ini}$ is the initial reservoir pressure (Pa).

The fracture permeability can be expressed as:


$$k_{f}=k_{f0}{\left(\frac{\varphi_{f}}{\varphi_{f0}}\right)}^{3}{\left(\frac{1-\varphi_{f0}}{1-\varphi_{f}}\right)}^{2}$$ (10)

The fracture width variation with porosity is given by:


$$w_{f}=w_{f0}-\Delta w_{f}=w_{f0}\left(\frac{1-\varphi_{f0}}{1-\varphi_{f}}\right)$$ (11)

Where $w_{f0}$ is the initial fracture width (m) and $\Delta w_{f}$ is the width reduction.

### 2.3. Geometric model and grid discretization

For the numerical simulations, a three-dimensional geological model of the shale gas reservoir was established, with dimensions of 2000 m × 255 m × 55 m. The grid size was set to 5 m in the horizontal, vertical, and transverse directions, resulting in 400 grids along the horizontal direction, 51 grids in the vertical direction, and 11 grids in the transverse direction. A horizontal well was positioned at the center of the model, with a total length of 1800 m, consisting of 10 fracturing stages and 6 fracture clusters per stage. The top and bottom boundaries of the model were defined as impermeable, while the outer boundaries were treated as closed boundaries.

**Fig 2:**
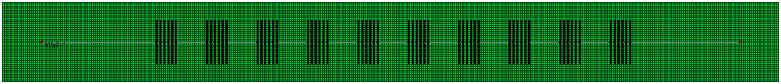
Schematic diagram of computational domain meshing (the horizontal well is 1800 m long, with 10 fracturing stages and 6 clusters per stage).

Fig 2 presents a schematic diagram of the computational domain meshing, clearly illustrating the spatial distribution of the multi-stage, multi-cluster hydraulic fractures along the horizontal well and the refined grid discretization.

### 2.4. Comparison and validation of numerical simulation with field results

In this study, the IMEX black oil simulator from CMG was used to simulate the dynamic production behavior of the shale gas reservoir, employing a dual-porosity/dual-permeability fluid flow model. This model accounts for the distinct flow behaviors in both the matrix and the fracture network, treating the matrix pores and fractures as separate yet interacting continua. The fracture network serves as a highly conductive pathway for gas migration, connecting the matrix to the wellbore and significantly enhancing overall productivity. The dual-porosity model thus better captures the coupled flow mechanisms in shale reservoirs, particularly in formations with well-developed fracture systems.

**Fig 3:**
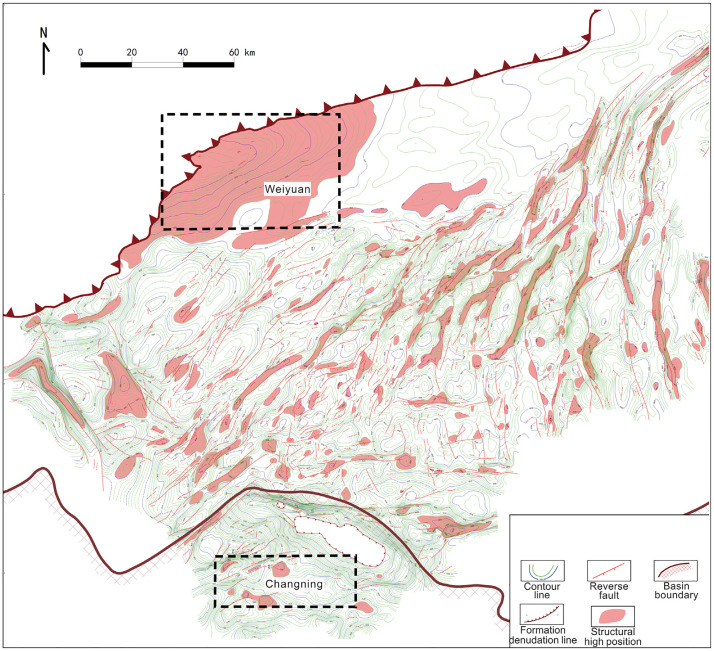
Location and regional division of the Changning–Weiyuan shale gas field in the Sichuan Basin, showing the Changning and Weiyuan blocks [19].

The Changning–Weiyuan shale gas field, located across Yibin and Neijiang cities in Sichuan Province, comprises the Changning and Weiyuan blocks, covering areas of 6844.977 km² and 6818.16 km², respectively (Fig 3) [19]. For model calibration, typical geological parameters of the Changning–Weiyuan demonstration area in the Sichuan Basin were adopted. This region is a representative area for commercial shale gas development in China, characterized by stable and thick pay zones, favorable geological conditions, and abundant resources.

The key input parameters for the model are presented in Tables 1 and 2. Table 1 lists the primary geological parameters of the Changning–Weiyuan demonstration area, including reservoir depth, porosity, permeability, fracture width, and pore pressure. Table 2 summarizes the fluid properties used in the simulation, such as matrix and fracture water saturation, Langmuir volume, and bottom-hole pressure. These parameters ensure that the numerical model accurately represents the actual operating conditions of the shale gas reservoir.

**Table 1:** Geological parameters of the Changning–Weiyuan demonstration area.

Parameter Name	Value	Parameter Name	Value
Burial depth/ m	3000	Natural fracture spacing/ m	40
Grid thickness/ m	5	Shale density/ kg/m³	2500
Natural fracture porosity	0.002	Reservoir temperature/ °C	80
Natural fracture permeability/ mD	0.7	Hydraulic fracture width/ m	0.025
Initial reservoir pressure/ kPa	30000	Hydraulic fracture permeability/ mD	5000
Initial natural fracture pressure/ kPa	30000	Rock compressibility/ 1/kPa	1.45e^-7^

**Table 2:** Fluid properties used in the simulation.

Parameter Name	Value
Natural fracture water saturation	0.8
Langmuir volume/ (m³/kg)	0.00272
Langmuir pressure/ kPa	4480
Reservoir matrix water saturation	0.25
Bottomhole pressure/ kPa	25000
Standard gas density/ kg/m³	0.58

The numerical model employed in this study was configured with a horizontal wellbore extending 1800 m in length, incorporating 13 fracturing stages spaced at 80 m intervals. Each stage contains 6 hydraulic fracture clusters with a spacing of 10 m. The hydraulic fractures extend vertically to 20 m and horizontally to 110 m. The reservoir matrix porosity was set to 6%, with a permeability of 0.0005 mD.

**Fig 4:**
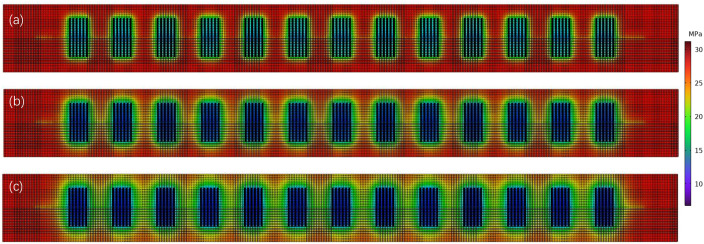
Reservoir pressure cloud maps of the shale gas reservoir after (a) 300 days, (b) 1000 days, and (c) 3000 days of production.

Based on the geological characteristics of the Changning–Weiyuan demonstration area, a numerical model was constructed to simulate the production dynamics of a horizontal well with a length of 1800 m. The evolution of the reservoir pressure field during production is shown in Fig 4. During production, a distinct low-pressure zone forms around the wellbore, creating a significant pressure difference with the far-field reservoir. The pressure distribution follows a clear trend: the pressure drop is greatest near the wellbore and gradually decreases with radial distance. As production continues, the low-pressure zone gradually expands, while the overall reservoir pressure declines.

When the reservoir pressure reaches the critical desorption pressure, the adsorbed gas begins to desorb in large quantities, providing a continuous gas supply for sustained production. The uniform distribution of the pressure gradient leads to higher gas flow velocities near the wellbore compared to the far-field reservoir, which directly impacts the ultimate cumulative production.

**Fig 5:**
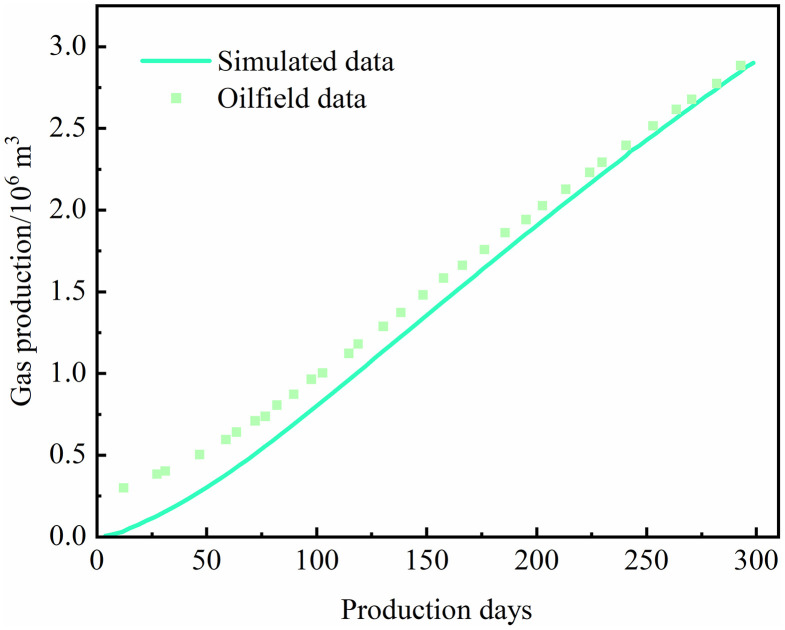
Comparison between model simulation and field data.

The comparison between the simulation results and field data, sourced from Qin [20], is presented in Fig 5. The simulated results exhibit the same trend as the field observations, confirming the feasibility and reliability of the model in capturing the coupled behavior of reservoir pressure evolution and gas production during shale gas development.

### 2.5. Design of the shale gas production capacity prediction dataset

The productivity of shale gas reservoirs is influenced by a variety of factors, including both engineering and geological parameters. To systematically investigate the impact and underlying mechanisms of these key factors on shale gas well production, a one-factor-at-a-time (OFAT) analysis approach was adopted. Under the condition that all other factors remain constant, the effects of key hydraulic fracturing parameters (such as the number of fracture clusters, cluster spacing, number of fracturing stages, and stage spacing) and reservoir physical properties (such as matrix porosity and permeability) were comprehensively studied.

By systematically varying these parameters, a complete numerical simulation scheme was developed to generate a high-quality dataset for training the deep learning production prediction model. The detailed simulation scenarios are summarized in Table 3.

**Table 3:** Numerical simulation schemes for shale gas production capacity.

Serial number	Cluster number	Cluster spacing/m	Stage number	Stage spacing/m	Fracture height/m	Fracture half-length/m	Porosity	Permeability/mD
1−1	**3**	10	10	60	20	110	0.06	0.0001
1-2	**6**	10	10	60	20	110	0.06	0.0001
1-3	**9**	10	10	60	20	110	0.06	0.0001
1-4	**12**	10	10	60	20	110	0.06	0.0001
2−1	6	**10**	10	80	20	110	0.06	0.0001
2−2	6	**15**	10	80	20	110	0.06	0.0001
2-3	6	**20**	10	80	20	110	0.06	0.0001
3−1	3	10	**10**	60	20	110	0.06	0.0001
3−2	3	10	**13**	60	20	110	0.06	0.0001
3−3	3	10	**16**	60	20	110	0.06	0.0001
3-4	3	10	**19**	60	20	110	0.06	0.0001
4−1	6	10	10	**40**	20	110	0.06	0.0001
4−2	6	10	10	**60**	20	110	0.06	0.0001
4−3	6	10	10	**80**	20	110	0.06	0.0001
4−4	6	10	10	**100**	20	110	0.06	0.0001
5−1	6	10	13	80	**10**	110	0.06	0.0001
5−2	6	10	13	80	**15**	110	0.06	0.0001
5−3	6	10	13	80	**20**	110	0.06	0.0001
5−4	6	10	13	80	**25**	110	0.06	0.0001
6−1	6	10	13	80	20	**50**	0.06	0.0001
6−2	6	10	13	80	20	**70**	0.06	0.0001
6−3	6	10	13	80	20	**90**	0.06	0.0001
6−4	6	10	13	80	20	**110**	0.06	0.0001
7−1	6	10	13	80	20	110	**0.05**	0.0001
7−2	6	10	13	80	20	110	**0.055**	0.0001
7−3	6	10	13	80	20	110	**0.06**	0.0001
7−4	6	10	13	80	20	110	**0.065**	0.0001
8−1	6	10	13	80	20	110	0.06	**0.0001**
8−2	6	10	13	80	20	110	0.06	**0.0005**

### 2.6. Data correlation analysis

In this study, the Pearson correlation coefficient was used to analyze the relationship between the design variables and the shale gas production capacity. The Pearson correlation coefficient measures the degree of linear association between two variables and is calculated as follows:


$$r=\frac{\sum \left(x_{i}-\bar{x})(y_{i}-\bar{y}\right)}{\sqrt{\sum {\left(x_{i}-\bar{x}\right)}^{2}\cdot \sum {\left(y_{i}-\bar{y}\right)}^{2}}}$$ (12)

where *x*_*i*_ and *y*_*i*_ represent paired observations, and $\bar{x}$ and $\bar{y}$ are the mean values of *x* and *y*, respectively. The correlation coefficient *r* ranges from −1–1, where **r* *= 1 indicates a perfect positive correlation, *r* = −1 indicates a perfect negative correlation, and *r* = 0 indicates no linear.

**Fig 6:**
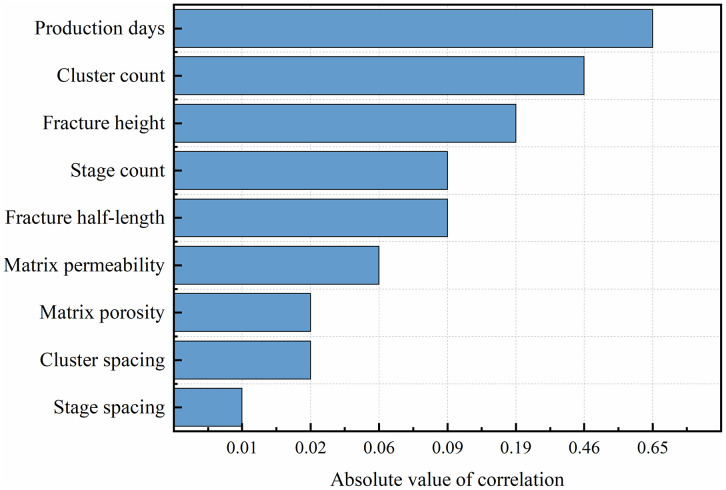
Ranking diagram of feature impact on target variables.

As shown in Fig 6, production days exhibit the highest absolute correlation coefficient with gas production (approximately 0.65), indicating that production duration is the dominant factor controlling shale gas yield. Longer production periods allow more gas to migrate from the matrix into the fracture network, resulting in higher cumulative output. The number of fracture clusters follows, with an absolute correlation value of about 0.45, suggesting that increasing the number of clusters enhances fracture connectivity and improves drainage efficiency. The fracture height shows a moderate correlation of approximately 0.20, implying that vertical fracture extension contributes to improved reservoir contact but with a relatively smaller impact compared to production time and cluster count.

In contrast, parameters such as fracture half-length and stage spacing exhibit weaker correlations, with absolute values around 0.09, indicating limited influence within the studied range. Meanwhile, matrix permeability, matrix porosity, and cluster spacing show very low correlation coefficients (|r| < 0.1), suggesting that their effects are secondary under the current simulation conditions. Overall, the correlation analysis identifies production time, cluster count, and fracture height as the most influential factors controlling shale gas production, which guided the selection of key input features for subsequent neural network modeling.

Based on the feature ranking diagram in Fig 6, it is evident that production days, the number of fracture clusters, and fracture height have the most significant impact on gas production. Therefore, in subsequent deep learning models, selecting the number of fracture clusters, fracture height, fracture half-length, and the number of fracturing stages as key input variables will help enhance the accuracy and reliability of the neural network in predicting cumulative shale gas production.

## 3. Shale gas production prediction model based on deep learning

In this study, two different types of neural network architectures (MLP and LSTM) were developed for shale gas production prediction. Based on the numerical simulation methods described earlier, daily gas production under various engineering parameter combinations was systematically calculated to establish a shale gas production prediction database. This database was then used to train and optimize the two neural network models, resulting in the corresponding intelligent production prediction models.

The MLP model was employed to capture the nonlinear relationships between geological and engineering parameters and shale gas production responses through a fully connected feed-forward architecture. In contrast, the LSTM network was designed to learn temporal dependencies in production data, effectively handling the sequential characteristics of shale gas output during different production stages. Such neural network frameworks have been successfully applied in subsurface characterization and well log prediction, demonstrating their robustness in modeling complex geoscientific datasets [21,22].

### 3.1. Data preprocessing

Based on the numerical simulation model established in Chapter 1, a dataset of shale gas production was constructed by systematically computing the production dynamics under different geological conditions and engineering parameter combinations. According to the correlation analysis results, four key influencing factors were selected, each with four different levels, resulting in 16 unique parameter combination scenarios. For each scenario, the daily production data over 3000 days of production were recorded.

**Fig 7:**
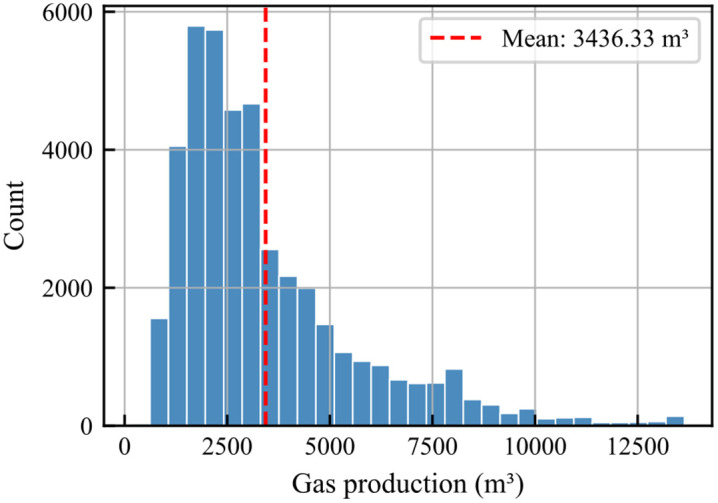
Distribution of daily shale gas production.

The numerical simulation yielded an initial dataset with 48,000 records. After removing duplicate entries, 42,000 unique samples of shale gas production dynamics were obtained. These samples cover the complete 3000-day production cycles across various parameter combinations, providing a diverse and robust dataset for training deep learning models. Fig 7 shows the distribution of daily shale gas production: the average daily production is 3436.33 m^3^, with a minimum of 169.68 m^3^; the 25th percentile is 1892 m^3^, the 50th percentile is 2763.38 m^3^, the 75th percentile is 4300 m^3^, and the maximum value is 13639.13 m^3^.

Given the relatively small numerical differences and the known range of the dataset, the min-max normalization method was applied for data standardization. This method, commonly used in deep learning preprocessing, preserves the relative relationships and distribution characteristics of the data. The normalization formula is expressed as:


$$x^{\prime }=\frac{x-\min(x)}{\max(x)-\min(x)}$$ (13)

where *x* is the original data, and max(*x*) and min(*x*)represent the maximum and minimum values of the dataset, respectively. This method can be applied to normalize both the input features and output labels for the deep learning model.

### 3.2. Shale gas production prediction model based on MLP

Energy is a crucial pillar for modern societal development. Building upon the numerical simulation dataset established earlier, a multi-layer perceptron (MLP) neural network model was developed to predict daily shale gas production. The model uses five key parameters—number of clusters, cluster spacing, number of stages, fracture height, and fracture half-length—as input features, and the daily gas production as the output variable, aiming to capture the complex nonlinear relationships under different parameter combinations.

To ensure sufficient expressiveness and generalizability, the MLP architecture comprises five layers: an input layer, three hidden layers, and an output layer. The input layer receives five-dimensional normalized feature vectors, while the three hidden layers contain 200, 200, 100, and 50 neurons, respectively. The ReLU activation function is employed in the hidden layers to accelerate convergence and improve the model’s nonlinear fitting ability. The output layer consists of a single neuron that directly predicts the daily shale gas production. Detailed network configurations are presented in Table 4.

**Table 4:** MLP network architecture.

Layer Type	Input Dimension	Output Dimension	Activation Function	Notes
Input	5	200	–	Input features after normalization/scaling
Hidden 1	200	200	ReLU	First hidden layer
Hidden 2	200	100	ReLU	Second hidden layer
Hidden 3	100	50	ReLU	Third hidden layer
Output	50	1	–	Predicted daily shale gas production

The model’s training objective is to minimize the mean squared error (MSE) [23] between the predicted and actual production values:


$$L(y,f(x))=\frac{\text{1}}{n}\sum_{i=1}^{n}{\left(y_{i}-f\left(x_{i}\right)\right)}^{2}$$ (14)

where $f\left(x_{i}\right)$ denotes the predicted value for the *i*-th sample, *y*_*i*_ represents the true production, and *n* is the number of training samples.

**Fig 8:**
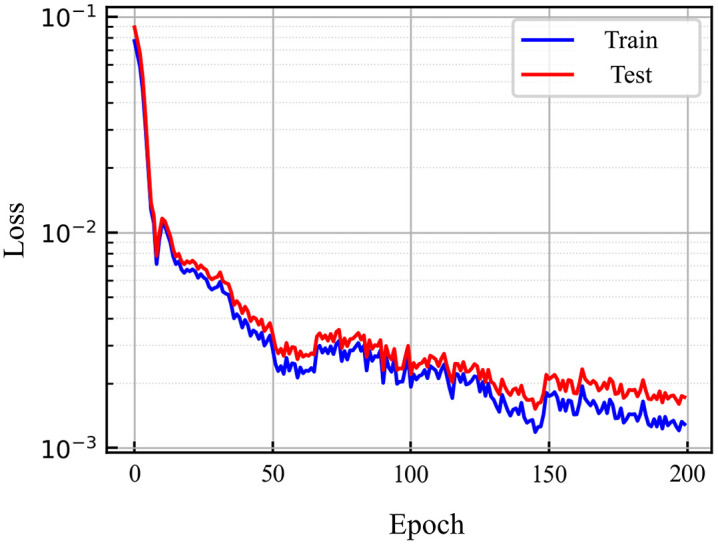
MLP model loss diagram.

The neural network model was implemented using the PyTorch framework. The dataset was randomly split into training and testing sets with an 8:2 ratio. Kaiming initialization was employed, ReLU [24] was chosen as the activation function, and the batch size was set to 32. The Adam [25] optimizer was used for 200 training epochs. After training, the MSE for the training set was 0.0026, and for the testing set it was 0.0030. Fig 8 illustrates the convergence of training and testing losses, indicating good convergence and generalization of the model.

**Fig 9:**
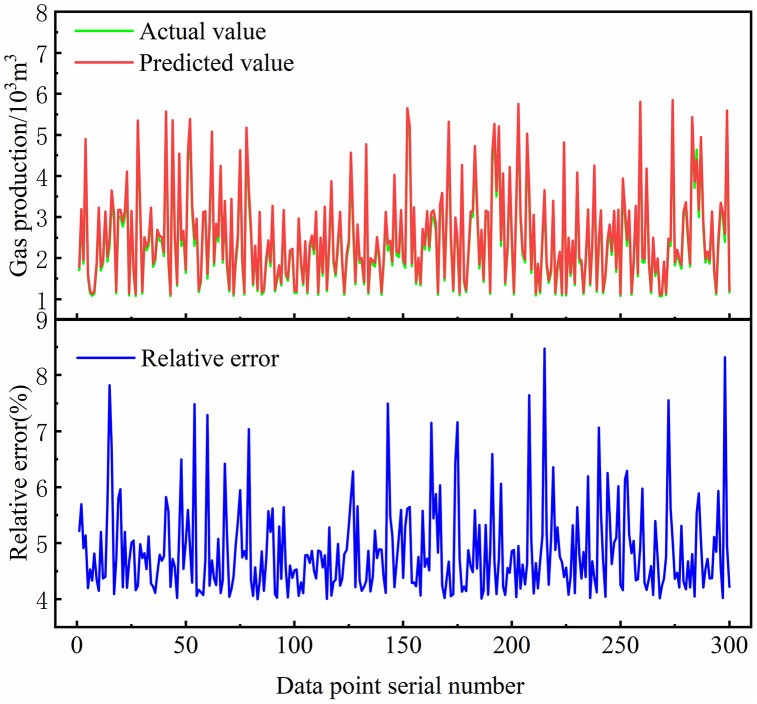
Comparison of MLP predicted values and actual values for daily shale gas production.

To further validate the accuracy of the MLP-based shale gas production prediction model, 300 testing samples were randomly selected. A comparison between the neural network predictions and actual observations is shown in Fig 9. The predicted production curve (blue) demonstrates a high level of consistency with the actual production curve (red), despite minor deviations at individual time points. Overall, the prediction errors remain within an acceptable range.

Error analysis indicates that the model can accurately capture production trends under various parameter combinations, demonstrating good generalization capabilities. Such predictive precision provides valuable insights for engineering applications and validates the reliability and applicability of the developed neural network model.

**Fig 10:**
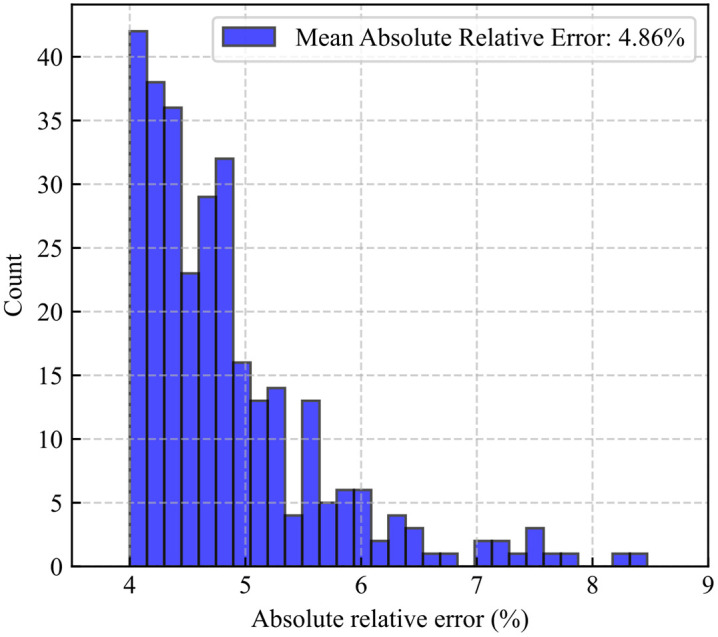
Distribution of relative errors in MLP predictions.

Fig 10 illustrates the distribution of relative errors between the neural network predictions and actual values for the 300 testing samples. The minimum relative error is 4.00%, the maximum relative error is 8.47%, and the average relative error is 4.86%. Most of the relative errors are concentrated in the range of 4%–6%, indicating that the model’s predictions are generally reliable. The error distribution approximates a normal distribution, with a peak occurring in the 4%–5% interval. This further confirms that the majority of prediction errors are small, meeting the requirements for engineering predictions. Only two data points exhibit relative errors greater than 8%, and these correspond to low daily production values, highlighting that the absolute errors are also minor.

### 3.3. Shale gas production prediction model based on LSTM

Recurrent Neural Networks (RNNs) are widely used for processing sequential data, with applications in natural language processing, time series analysis, and speech recognition. However, traditional RNNs often suffer from vanishing and exploding gradients when dealing with long sequences, making it challenging to capture long-term dependencies. To address this issue, Long Short-Term Memory (LSTM) networks were introduced. By incorporating memory cells and gating mechanisms (input, forget, and output gates), LSTMs effectively model long-range dependencies within sequences [26].

In this study, a two-layer LSTM model was developed to predict daily shale gas production. The architecture is as follows: the first LSTM layer contains 100 neurons and is configured with return_sequences = True to output the complete time series (input shape: time steps = 2, features = 5). L2 regularization (coefficient 0.01) is applied to prevent overfitting, followed by a Dropout layer (dropout rate 0.2) to further reduce overfitting. The second LSTM layer also has 100 neurons but uses return_sequences = False to output only the final time step’s result. It also employs L2 regularization and another Dropout layer [27] (dropout rate 0.2). Finally, a fully connected Dense layer predicts the daily gas production value. The model uses mean squared error (MSE) as the loss function and is optimized with Adam, using MSE as the performance metric.

**Fig 11:**
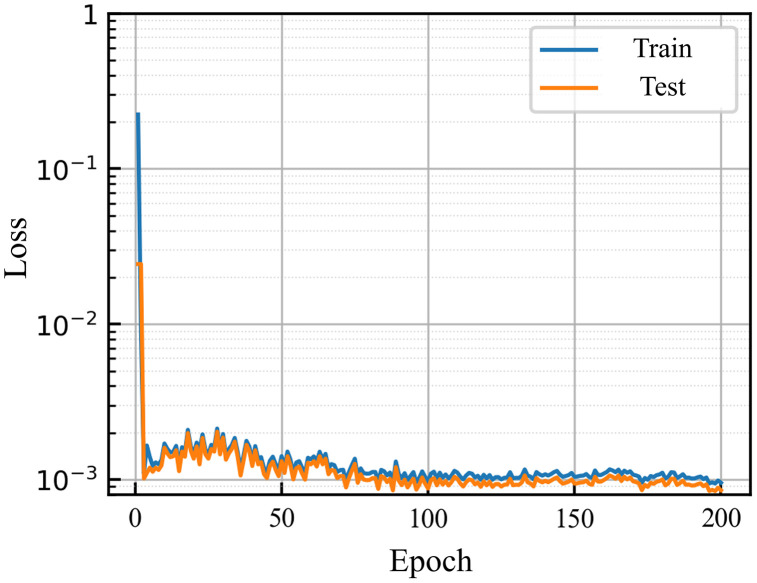
LSTM model loss diagram.

The training and testing loss curves of the LSTM model are shown in Fig 11. The results indicate that the LSTM network achieves good convergence during shale gas production prediction, with loss values converging to the order of 10^-3, suggesting a high prediction accuracy.

**Fig 12:**
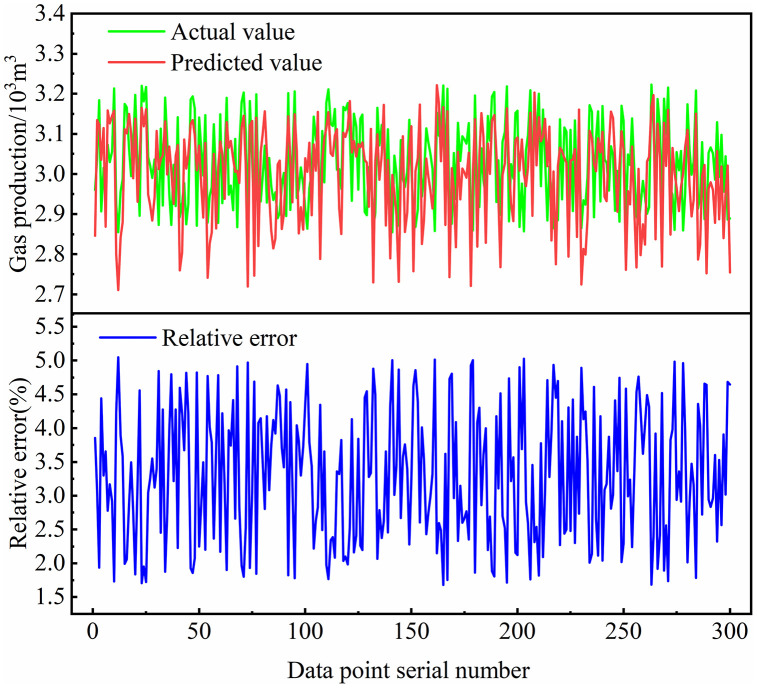
Comparison of LSTM predicted values and actual values for daily shale gas production.

Similarly, to further validate the accuracy of the LSTM-based shale gas production prediction model, 300 testing samples with different parameter combinations were randomly selected. The neural network predictions of daily gas production were compared with the actual values. Fig 12 shows the comparison between the LSTM predicted values and actual daily shale gas production. As seen in the figure, the predicted and actual values align well overall, although slightly less so than in the MLP model. This is mainly because the randomly selected daily production data lie within a relatively stable range, resulting in overlapping curves.

**Fig 13:**
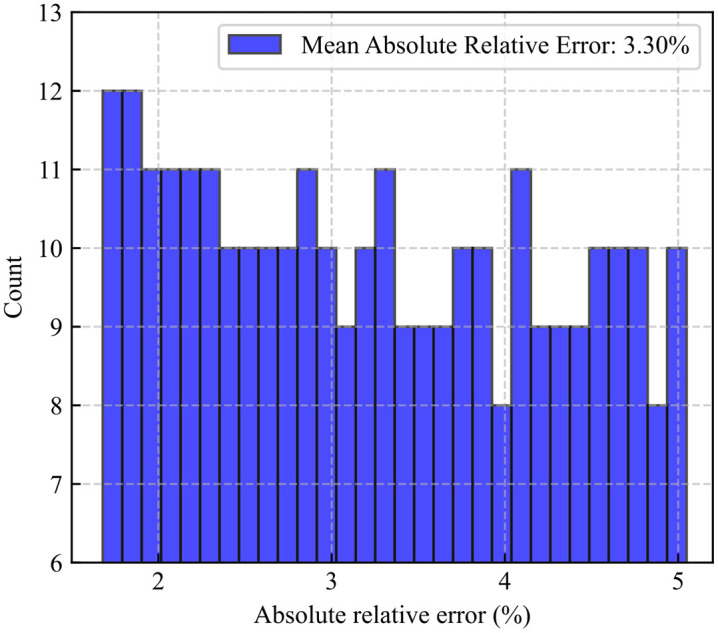
Distribution of relative errors in LSTM predictions.

Fig 13 presents the distribution of relative errors between the LSTM predictions and actual values for the 300 testing samples. The minimum relative error is 1.67%, the maximum is 5.04%, and the average is 3.30%. The relative errors are most frequently distributed in the 0%–2.5% interval, indicating that the model’s prediction errors are generally small and the results are reliable, meeting the requirements for engineering predictions.

## 4. Results and discussion

To evaluate the predictive performance of the two neural network models, three sets of parameter combinations were selected for comparison between the predicted and actual gas production rates. The parameter settings were as follows:

(1) Example 1: 6 fracturing clusters, a fracture half-length of 110 m, 10 fracturing stages, and a fracture height of 20 m;
(2) Example 2: 6 fracturing clusters, a fracture half-length of 70 m, 10 fracturing stages, and a fracture height of 20 m;
(3) Example 3: 6 fracturing clusters, a fracture half-length of 70 m, 10 fracturing stages, and a fracture height of 25 m.

**Fig 14:**
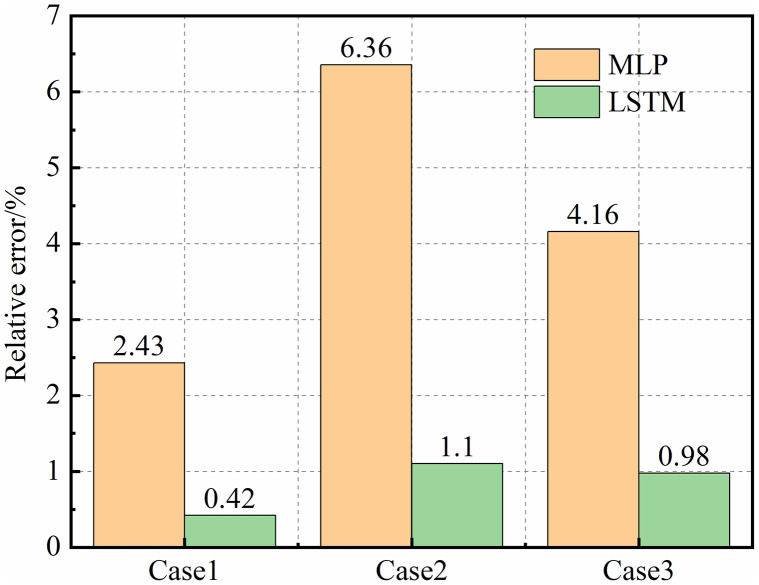
Comparison of relative errors between predicted and actual values for different models (based on the 1000th day data of three example cases).

The predicted and actual production rates on the 1000th production day were used for comparative analysis.As shown in Fig 14, the relative errors between the predicted and actual gas production for the MLP and LSTM models were as follows:

(1) For the MLP model: 2.43%, 6.36%, and 4.16%, respectively.
(2) For the LSTM model: 0.42%, 1.10%, and 0.98%, respectively.

It is evident that the LSTM model consistently outperforms the MLP model across all parameter combinations, achieving relative errors below 1.1%. This improvement demonstrates the strong capability of LSTM to learn complex temporal patterns in shale gas production data. The superior performance of the LSTM network arises from its gating mechanisms (forget, input, and output gates), which enable the model to capture long-term dependencies and sequential correlations that are intrinsic to production time series. In contrast, the MLP, as a static feed-forward neural network, lacks temporal memory and therefore cannot effectively model the dynamic evolution of production rates over time.

Shale gas production is inherently nonlinear and time-dependent, typically exhibiting an initial rapid decline followed by a gradual stabilization phase. The cell-state structure of the LSTM allows the model to preserve and update critical features across multiple time scales, which is essential for representing both the short-term pressure response immediately after fracturing and the long-term depletion trend during sustained production. Although the MLP can effectively map nonlinear relationships between static parameters, it overlooks the sequential dependencies in production behavior, leading to higher prediction errors and limited temporal adaptability.

Overall, these results highlight the importance of temporal modeling in improving predictive reliability for shale gas production forecasting. The LSTM-based deep learning approach not only achieves lower overall error but also better reflects the physical processes of pressure evolution, reservoir depletion, and production stabilization. Compared with the traditional MLP-based machine learning model, the LSTM exhibits stronger robustness, adaptability, and generalization capability, making it more suitable for practical field-scale applications where cumulative effects and time-varying production dynamics play a dominant role.

## 5. Conclusions

This study systematically investigated shale gas production prediction by integrating numerical simulation and deep learning approaches, leading to the following conclusions:

(1) A comprehensive database of daily shale gas production under various engineering parameter combinations was constructed using numerical simulation, providing high-quality and diverse samples for training and validating deep learning models.
(2) Among the deep learning models evaluated, the LSTM neural network outperformed the MLP model in prediction accuracy and generalization, demonstrating a better ability to capture the dynamic temporal features of shale gas production data and confirming its applicability in production forecasting.
(3) Future work may involve integrating real field monitoring data with numerical simulation results and further optimizing the deep learning model architecture to enhance its engineering adaptability and predictive power, thereby supporting the efficient development of unconventional shale gas reservoirs.

## Supporting information

S1 File shale_gas_data. (XLSX)
